# Assessing suitability for long-term colorectal cancer shared care: a scenario-based qualitative study

**DOI:** 10.1186/s12875-020-01311-w

**Published:** 2020-11-21

**Authors:** Kylie Vuong, Kerry Uebel, Maria Agaliotis, Stella Jun, Jane Taggart, Sue Suchy, Winston Liauw, Melvin Chin, Kate Webber, Mark Harris

**Affiliations:** 1 School of Population Health, University of New South Wales, Sydney, Australia; 2 Australian Institute of Health Service Management, University of Tasmania, Sydney, Australia; 3 Translational Cancer Research Network, University of New South Wales, Sydney, Australia; 4 Centre for Primary Health Care and Equity, University of New South Wales, Sydney, Australia; 5 Saint George and Sutherland Clinical School, University of New South Wales, Sydney, Australia; 6 Prince of Wales Clinical School, University of New South Wales, Sydney, Australia; 7 School of Clinical Sciences, Monash University, Clayton, Australia; 8 Department of Medical Oncology, Monash Health, Clayton, Australia

**Keywords:** Capacity building, Colorectal neoplasms, Health communication, Health services, Primary health care, Survivorship

## Abstract

**Background:**

Shared care is the preferred model for long-term survivorship care by cancer survivors, general practitioners and specialists. However, survivorship care remains specialist-led. A risk-stratified approach has been proposed to select suitable patients for long-term shared care after survivors have completed adjuvant cancer treatment. This study aims to use patient scenarios to explore views on patient suitability for long-term colorectal cancer shared care across the risk spectrum from survivors, general practitioners and specialists.

**Methods:**

Participants completed a brief questionnaire assessing demographics and clinical issues before a semi-structured in-depth interview. The interviews focused on the participant’s view on suitability for long term cancer shared care, challenges and facilitators in delivering it and resources that would be helpful. We conducted thematic analysis using an inductive approach to discover new concepts and themes.

**Results:**

Interviews were conducted with 10 cancer survivors, 6 general practitioners and 9 cancer specialists. The main themes that emerged were patient-centredness, team resilience underlined by mutual trust and stronger system supports by way of cancer-specific training, survivorship care protocols, shared information systems, care coordination and navigational supports.

**Conclusions:**

Decisions on the appropriateness of this model for patients need to be made collaboratively with cancer survivors, considering their trust and relationship with their general practitioners and the support they need. Further research on improving mutual trust and operationalising support systems would assist in the integration of shared survivorship care.

**Supplementary Information:**

The online version contains supplementary material available at 10.1186/s12875-020-01311-w.

## Background

Cancer survivors, people who have experienced cancer, are a growing proportion of the population, representing 4.6% of the Australian population in 2014 and this is expected to continue to rise [1–3]. Cancer survivorship care includes the monitoring of cancer recurrence, as well as preventive care, management of comorbidities, and psychosocial support. In Australia and internationally, the conventional model of cancer survivorship care is mainly specialist-led, with cancer survivors continuing to attend their cancer specialists long-term and relatively smaller contributions from primary care [4].

Primary care focuses on whole person care, continuity of care and comprehensiveness [5]. These core features are particularly beneficial in cancer survivorship care and more involvement from primary care has been extensively promoted by governments, health funders and discipline leaders [6, 7]. Shared care is a system of care that involves both primary care and specialist services working collaboratively in partnership to provide patient-centred cancer care using agreed processes and outputs [3].

Recent systematic reviews have found equivalent quality of life, mental health and clinical outcomes for cancer survivors who follow-up in primary care and specialists services [8], with lower costs associated with shared care models [9]. It is also the preferred model of survivorship care by cancer survivors, general practitioners and specialists [10]. However, survivorship care remains specialist-led and the involvement of general practitioners continues to be both poorly structured and unsupported [11]. The role of general practice in cancer follow-up is not well defined and there is substantial variation on both cancer survivorship guidelines and practice in primary care [12]. Previous research by our team and others have highlighted concerns over primary care’s cancer-specific expertise and interest, time pressures in primary care, and survivors’ timely access to cancer services [10, 13].

As a way forward, a risk-stratified approach based on the: (1) type of cancer, (2) effects of treatment, (3) co-morbidities, (4) the patient’s ability to manage and (5) required level of health professional involvement has been proposed to identify low-risk patients for early re-engagement with primary care services in long-term shared care after survivors have completed adjuvant cancer treatment [14]. Before the translation of this approach into practice, we need to understand the views on patient suitability for cancer shared care from survivors, general practitioners and specialists.

### Decision support tool and colorectal cancer survivor scenarios

To assist in the identification of colorectal cancer survivors suitable for long-term shared care, a decision support tool was developed using an iterative process to reach expert consensus based on patient indicators that are easily obtainable in the clinical setting. The expert panel included: cancer survivors (JL and SS), general practitioners (KV and MH) and cancer oncologists (KW and MC). The final patient indicators associated with low risk of complications included the absence of: (1) oxaliplatin chemotherapy; (2) bowel incontinence after treatment; and (3) stoma after treatment. Based on these indicators, we developed three colorectal cancer patient scenarios: (1) a 55 year old with stage II colorectal cancer, treated without oxaliplatin chemotherapy, with no bowel incontinence and no stoma, representing survivors at low risk of complications; (2) a 75 year old with rectal cancer, treated with oxaliplatin chemotherapy and with a stoma, representing survivors at moderate risk of complications; and (3) a 43 year old with Lynch syndrome and stage II colorectal cancer, treated with oxaliplatin chemotherapy and with occasional bowel incontinence, representing survivors at moderate risk of complications and high risk of recurrence (see Supplementary).

This pre-implementation study aims to use patient scenarios to explore views on patient suitability for long-term colorectal cancer shared care across the risk spectrum from survivors, general practitioners and specialists.

## Methods

Qualitative methods and results are reported in accordance with the consolidated criteria for qualitative research [15].

### Participants

Colorectal cancer survivors, general practitioners and cancer specialists were purposefully sampled using a maximum variation technique to select participants from both genders and different age groups [16] from 12 November 2018 to 5 June 2019. Survivors were recruited from the participating cancer specialists and eligible if they had completed definitive primary treatment, were in remission and well enough to participate. General practitioners were recruited from the Central and Eastern Sydney Primary Health Network, New South Wales, Australia [17]. Cancer specialists, including oncologists, surgeons and nurse coordinators, from colorectal cancer multidisciplinary teams were recruited from the South-Eastern Sydney Local Health District, New South Wales, Australia [18], through cancer services using snowball sampling, with participants providing the names and contacts of potential participants. The geographical catchment of the Central and Eastern Sydney Primary Health Network [17] closely aligns with that of the South-Eastern Sydney Local Health District [18]. General practitioners and cancer specialists were eligible if they were clinically active.

### Data collection

**Fig. 1:**
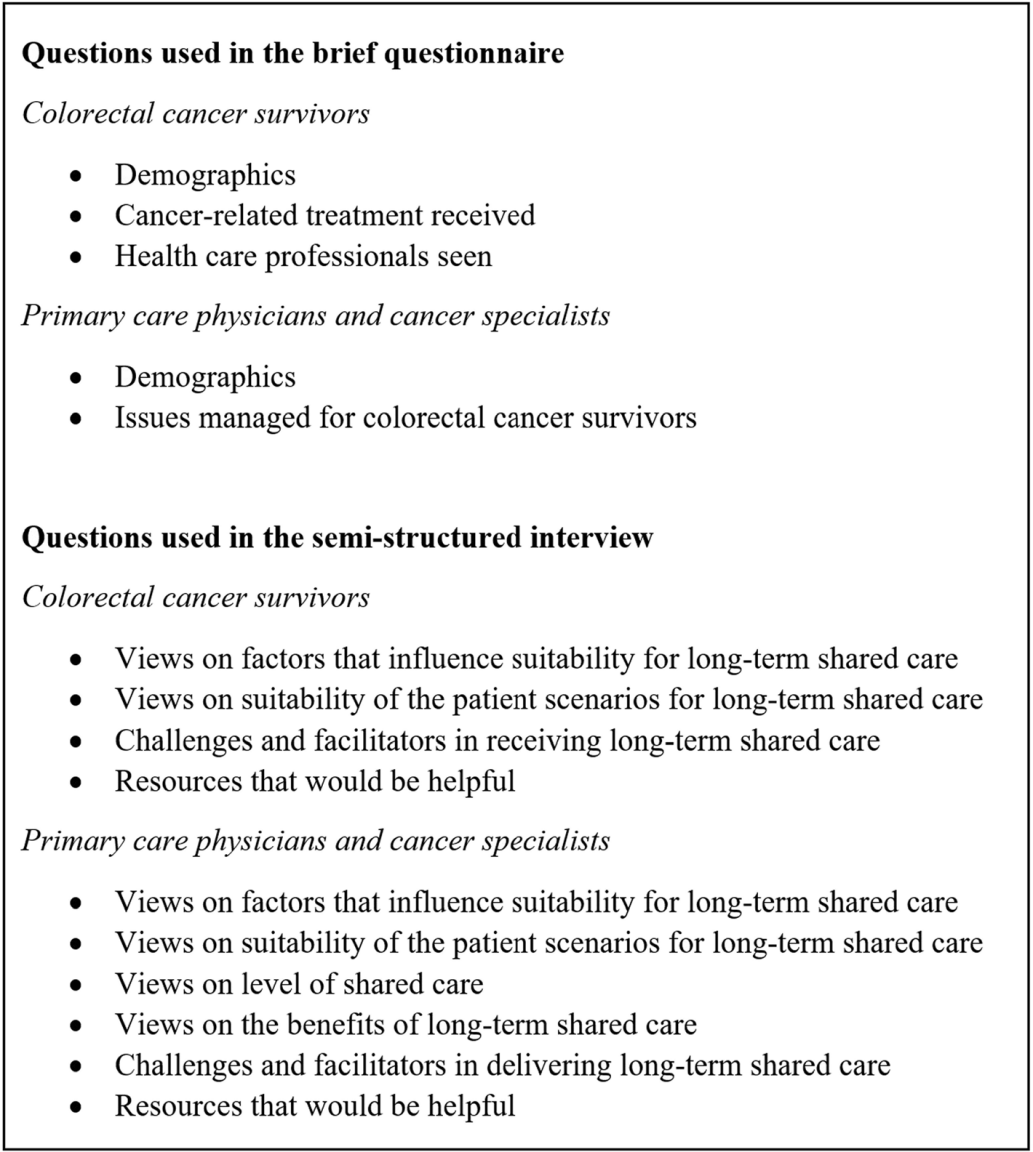
Outline of questions. This figure provides an outline of the questions used in the brief questionnaire and semi-structured interview.

Consented participants completed a questionnaire on demographic information and colorectal cancer experiences (see Supplementary) before a single semi-structured in-depth interview with a researcher (KU, a general practitioner with research experience in chronic disease management; MA, a health service researcher with a background in clinical nursing; or SJ, a health service researcher). The interviewers had no prior relationships with the participants. Participants were sent copies of the patient scenarios and were given time to read them immediately before the interview. The interviews were guided by a schedule (see Fig. 1) and were conducted with value-neutrality. They were audio-recorded, professionally transcribed verbatim and de-identified. Transcripts were not returned to participants. Participants were offered an AUD$80 shopping voucher for the time taken to complete the interview. Ethical approval (No. 18/082) was obtained from the South Eastern Sydney Local Health District Human Research Ethics Committee, and informed written consent was obtained from all participants.

### Data analyses and interpretation

Thematic analysis was conducted following steps suggested by Braun and Clarke [19] using an inductive approach to discover new concepts and themes. To ensure comprehensiveness, all interview transcripts were read and re-read by three researchers (KV, KU and MA), who independently identified the initial codes and clustered them into categories and themes. The three researchers met frequently to revise and further refine the codes, categories and themes by discussing similarities, differences and contradictions. To strengthen the finding’s confirmability [20], there were regular discussions with other members of the research team (SS, a cancer survivor; and MH, a general practitioner with research experience in chronic disease).

Analysis was focused on the individual interview transcripts before looking at themes across all the transcripts, with attention to the similarities and differences in perspectives between cancer survivors, general practitioners and cancer specialists. A systematic analytical audit trail was kept. QSR NVivo12 software was used to code and manage the interview data.

## Results

Of 33 potential participants approached, 26 agreed to participate. One cancer specialist participant was excluded because they were not clinically active in care of patients with colorectal cancer. We conducted 23 face-to-face interviews and two telephone interviews with the participants; the interviews took on average 40 min to complete. Participant characteristics are shown in Table 1.

**Table 1:** Participant characteristics 2018–2019

	Patient	General practitioner	Cancer specialist^a^
Sex			
Female	5	4	5
Male	5	2	4
Age			
< 29			1
30–39		2	2
40–49		1	3
50–59	2	2	2
60–69	4	1	1
≥ 70	4		
Age at colorectal cancer diagnosis			
50–59	2	Not applicable	Not applicable
60–69	6		
≥ 70	2		
Years in practice			
0–4	Not applicable	2	1
5–9			3
10–14		1	3
15–19			1
20–24		1	
≥ 25		2	1
Number of half-day sessions worked per week			
≤ 10	Not applicable	4	9
> 10		2	
Number of patients with a history of colorectal cancer seen in last 12 months			
≤ 10	Not applicable	4	9
> 10		2	

^a^Cancer specialists included six oncologist, one surgeon and two nurse coordinators

Overall, long-term shared care was perceived to be beneficial to the patient, health care providers and the health system. All patient scenarios presented were perceived to be suitable. We identified three main themes that influence patient suitability: patient-centred factors, team-based factors and availability of supporting infrastructure and resources (Table 2). The views of patients, general practitioners and specialists were different across some elements.

**Table 2:** Summary of the main themes and categories from the interviews

Themes associated with suitability for long-term shared cancer care	Categories
Patient-related factors	Physical care needs
	Psychosocial care needs
Team-resilience	Patients trusting physicians
	Physicians trusting patients
System supports	Cancer-specific training
	Survivorship care plans
	Shared information systems
	Care coordination and navigation support

### The benefits of shared care

All participants felt long-term shared care was beneficial. It was perceived to be personally rewarding and that it provided more resources, with potential to provide more holistic patient care.

*As a cancer patient, I rely on my GP very much…even when the doctor in the cancer centre recommend me to take some medicine or whatever, I go back to my GP and ask his opinion (Patient 6)*

*…a lot of people rely on a GP for a lot of emotional support- and the specialist can’t give that every 6 months or 12 months. (Patient 10)*

*…especially for those non-English speaking background patients. I think it’s very helpful, and they have a lot of trust in their GPs…. (General practitioner 1)*

*I think it's an opportunity for them to engage and actually to have better attention to all these other issues. (Specialist 8)*

### Patient-centredness

Patient-centred care is the preferred approach to delivering health care and is embedded within the paradigm of holistic care. This approach emphasises the need to provide coordinated health care, encompassing physical and psychosocial care, in partnership with the patient and their family that is inclusive of their preferences [21]. This attitude resonated among all the cancer survivors, general practitioners and cancer specialist interviewed. Whilst there was consensus among participants that all scenarios were “suitable to some degree”, there was also endorsement that “it has got to be a choice”.

All participant groups felt that colorectal cancer survivors with multiple co-morbidities would be suitable for more long-term shared care.

*…the GP would be ideal for the rest of his problems. (Patient 3)*

*…they [GPs] really know them well, they know their comorbidities well, they know all their medications well…they know their psycho-social issues a lot more, they are probably much more equipped than me (Specialist 2)*

Patient and specialist participants felt cancer survivors with high cancer recurrence risk, complications from treatment, multiple cancer sites and changing treatment plans, such as the 43 year old patient with Lynch syndrome (Supplementary, Patient scenario 3), would need more reviews with the cancer specialists and may be less suitable for long-term shared care.

*…where there’s a high risk that aren’t, you know, stable, then that’s a different story. (Patient 1)*

*I think the neuropathies, even if you’re oncologist, can be difficult to treat, some of it is reversable over time (Specialist 4)*

*We like to see the stoma more regularly… (Specialist 7)*

There were contrasting views about long-term cancer shared care depending on whether the patient was more anxious about missing out on specialist cancer care or having to present to a cancer service in hospital with all its associations. Some participants identified patients who had high anxiety about their cancer would not be suitable for long-term shared care.

*…after I’m on the yearly monitoring...then the GP would be fine,… even now, although I don’t have huge problems, I would like to see the oncologist…it’s a very confronting thing to have cancer, and I don’t know how an individual will handle it and how they will see a GP after going through all that. You see, you put your trust in your own oncologist. (Patient 3)*

On the other hand, participants also felt that patients with anxiety about attending cancer services in hospitals would benefit from long-term shared care.

*…the hospital becomes an anxiety-provoking remembrance so they can be worse (Specialist 6)*

### Team resilience underlined by mutual trust

The participants described working team relationships based on mutual trust as an important factor in determining suitability for shared care. Patients and specialists identified having a regular general practitioner that they trust as a pre-requisite for long-term shared care.

*we’ve developed a relationship for over 10 years or – I forgot how long exactly, but have this trust in between us… (Patient 6)*

*I think a lot of that hinges on the oncologist, but also who the general practitioner is…(Specialist 4)*

However, for some patients, trust in the specialist is also a reason for not wishing to participate in long-term shared care.

*you put your trust in your own oncologist. He takes you step-by-step at the beginning to explain everything. You spend hours in the room and he goes through it, he gives you all the information that you read and you feel safe that you’ve got someone who knows what they’re talking about, your life is in their hands. (Patient 3)*

Patients were more likely to trust their general practitioners and specialists if they were perceived to have the knowledge and time to provide care.

*You know how busy GPs are too. Do they get some special training or are they seeing you as their normal patient? (Patient 5)*

*I hate going to him [oncologist], because you’ve got to wait an hour every time you go there, you make an appointment, you wait and, oh, he’s busy. (Patient 9)*

For general practitioners and specialists, the “implied trust across the whole network” is important. General practitioners and specialists were more likely to encourage patients to consider long-term shared care if they were perceived to be proactive in their care

*patients that are going to come back, motivated, and stuff like that. I mean they're suitable for either model (General practitioner 5)*

*So they need to understand the importance of why they're going to the GP at that particular time. (Specialist 1)*

### System supports to promote long-term cancer shared care

Whilst there was consensus on the suitability and benefits of long-term shared care, the participating general practitioners and specialists had reservations in implementing long-term shared care in the current setting due to the difficulties in fast tracking patients back to cancer services, poor communication systems between primary and tertiary care, and low renumeration.

*It’s really hard, and then I know there are always different specialists. The on-call person will have no idea about my patient, they have to read the [electronic records] and then trying to figure it out. (General practitioner 1)*

*there can be real delays in letters being typed and going out. I have had patients die before letters get to the GPs (Specialist 2)*

This feeling was more pronounced among general practitioners compared with specialists.

*…it's really hard to remember (that) the GP’s part of the team. (General practitioner 2)*

Effective long-term shared cancer care should include: clear protocols for cancer follow up care that is documented in an individualised survivorship care plan, cancer-specific training to address perceived knowledge deficits in interested general practitioners, shared information systems and fast-track measures back into the hospital system when patients were unwell. Shared health records, either in paper format, like the cards used in antenatal shared care, or electronic format, were suggested as possibilities for improving team communication.

*I think it needs to have all his follow ups that are required, very clearly written and clear about who’s going to do that, about whose responsibility are what, and in what timeframes. (Patient 1)*

*I'm happy managing, or most of us are happy managing anyone as long as there's a set plan, and if there is a problem along that they can be seen – like they can be fast-tracked back to see the team. (General practitioner 3)*

Participants identified the importance of cancer follow up care coordination with either primary care or cancer services taking the lead, and navigational support from key individuals such as a care coordinator.

*I’d bring the GP into the team from day 1, that way I can test their level of interest and involvement throughout the process. (Specialist 3)*

## Discussion

Ours is the first study to explore views on the suitability of patients for long-term shared care among colorectal cancer survivors, general practitioners and specialists using risk-stratified patient scenarios. There was consensus among both patient and physician participants that some shared cancer care arrangement was potentially beneficial to all colorectal cancer survivors. Whilst a risk-stratified approach to cancer shared care has been proposed by national and international organisations [7, 14], findings from our pre-implementation study suggests that all colorectal cancer survivors would be suitable conditional on patient-centred and team-based factors, and adequate supports being available for effective shared care.

Patient-centred factors such as cancer survivors with higher cancer recurrence risk, complications from treatment, multiple cancer sites and changing treatment plans would benefit more from specialist reviews, but these should not prohibit the patient from participating in long term shared care. The importance of incorporating the preferences of cancer survivors into follow up care planning has also been found in previous studies and is the recommended approach to patient care [13, 21–23]. Lawn and colleagues [13], through a community forum, similarly found strong desire for cancer survivors to be placed at the centre of their care to improve ownership and ensure accurate communication between the team members. However, as in our study, variations were reported between individual cancer survivors, and between cancer survivors and physicians on the degree of patient involvement in a shared model of cancer care [22, 23].

Trust is “the willingness of a party to be vulnerable to the actions of another party based on the expectation that the other will perform a particular action important to the trustor, irrespective of the ability to monitor or control that other party” [24]. Mutual trust builds team resilience and is an essential component of the patient-doctor relationship. It has positive consequences in diagnoses, satisfaction, patient adherence to treatment and continuity of care [25]. In our study, patients perceived the physician’s cancer-specific knowledge, commitment and level of communication as measures of trust. The physicians perceived the patient’s level of motivation, responsibility and understanding as measures of trust. Whilst a large body of literature exists on improving patients’ trust in physicians, in an era of increasing patient autonomy and shared models of care, physicians must also trust their patients and recognise the measures of trust that are important for them. However, there have been a few studies conducted on improving mutual trust in the patient-doctor relationship [25–28]. A review by Wilk and Platt found 446 articles on physicians and trust in the general population, of these 30 articles mentioned the physicians’ trust in patients [26]. Wilk and Platt identified themes on perceived patient deception, drug-seeking behaviours, miscommunications, which we did not identify in our interviews with general practitioners and cancer specialists [26].

Most participants felt that there was a lack of support systems to implement long-term shared care, and this feeling was more pronounced among general practitioners. As in previous studies, there were concerns about barriers to fast tracking patients with complications or possible recurrence back to cancer services, poor communication systems between primary and tertiary care, and low renumeration for time invested [10, 13, 22, 29, 30]. This study also provided multiple examples of the participants’ resourcefulness with proposed strategies to promote shared care such as cancer-specific training to address perceived knowledge deficits in interested general practitioners, clear protocols for cancer follow up care that is documented in a survivorship care plan, shared information systems, fast-track measures back into the hospital system when patients were unwell, improved care coordination and navigational supports. The shift to telehealth that has been precipitated by the COVID-19 pandemic [31] means long-term shared care may be more feasible and accessible, with cancer specialists now able to extend their support to survivors and general practitioners via telehealth.

It is a strength of our study that the cancer survivors and specialists were linked, which meant the discussions on cancer care systems were not abstract. Similarly, the general practitioners and specialists work in an overlapping geographical area.

This study also has several potential limitations. The sample was small. However, our participants included both genders from different age groups that reflected the diversity from the geographical catchment of the Central and Eastern Sydney Primary Health Network [17] and the South-Eastern Sydney Local Health District [18], and we managed to reach data saturation. Whilst we were able to capture views from a heterogeneous group, it may not reflect the views of patients and physicians in other settings, especially rural and remote settings. In this pre-implementation study, we used patient scenarios across the risk spectrum to explore views on patient suitability for long-term colorectal cancer shared care instead of prospective real-world clinical data. While it is possible that the participants’ view on suitability for shared care may differ in the real-world, previous studies comparing simulation of clinical encounters and real data have shown similar decisions and outcomes [32, 33].

## Conclusions

The findings from our study suggest that all colorectal cancer survivors would be suitable for long-term shared care, conditional on patient-centred factors, mutual trust, and the availability of system supports. Further research on improving mutual trust and operationalising supports systems could lead to more integrated survivorship care models.

## Supplementary Information

**Additional file 1: Supplementary 1.** Patient scenarios. **Supplementary 2.** Questionnaire.

## Data Availability

The datasets generated and analysed during the current study are not publicly available due to privacy concerns but are available from the corresponding author on reasonable request subject to approval from South Eastern Sydney Local Health District Human Research Ethics Committee.
