# Supplementary material for: Assessing suitability for long-term colorectal cancer shared care: a scenario-based qualitative study
Source: BMC Fam Pract. 2020 Nov 21;21:240. doi: 10.1186/s12875-020-01311-w (PMC7680065; doi:10.1186/s12875-020-01311-w)
Supplement: Supplementary file 1 — Additional file 1: Supplementary 1. Patient scenarios. Supplementary 2. Questionnaire. [file 12875_2020_1311_MOESM1_ESM.docx]

**Supplementary**

**Supplementary 1: Patient scenarios**

**Patient scenario 1**

- 55 year old woman, diagnosed with stage II (node negative) colorectal cancer 12 months ago after presenting with rectal bleeding
- Hemicolectomy, adjuvant chemotherapy with 5-fluorouracil, completed 4 months ago
- During chemo: hand-foot syndrome, this has resolved
- Ongoing issues:

1. Fatigue – limiting capacity to get back to previous work or exercise at recommended levels
2. Anxiety – saw psychologist a few times during chemo but not since
3. Sleep disturbance
4. Neurocognitive impairment
5. Occasional diarrhoea, related to diet

- Current follow up with medical oncology team 3 monthly and surgeon annually
- Comorbidities: menopausal symptoms (pre-date diagnosis and unchanged), osteopenia
- Ex-smoker, has cut down on alcohol use at diagnosis (now 1-2 glasses wine, 2-3 days/week)
- Works as receptionist, divorced with difficult family circumstances
- Never attended for colorectal, breast or cervical cancer screening.

In plain English

Mary, a 55 year old woman, had bowel cancer 1 year ago after blood was found in her stools. The bowel cancer grew through the muscle in the bowel, but did not spread to the lymph nodes. Half of her bowel was removed; she then had chemotherapy. She had redness, swelling and pain in the palms of her hand and soles of her feet during the chemotherapy, this is no longer a problem. However, she is still very tired and unable to get back to work, anxious, unable to sleep and has memory loss. She also gets diarrhoea if she is not careful with her diet.

She has pre-menopausal signs including hot flushes, and has low bone density (osteopenia).

She works as a receptionist and is recently divorced. She is an ex-smoker and cut her alcohol down to 1-2 glasses of red wine 2-3 days per week. She sees the surgeon once yearly and medical oncology team 3 monthly.

**Patient scenario 2**

- 75 year old man, diagnosed with node positive rectal cancer 2 years ago after asymptomatic iron deficiency found on routine bloods with general practitioner
- Neoadjuvant chemoradiation with capecitabine
- AP resection with permanent stoma
- Adjuvant chemotherapy with FOLFOX (5-fluorouracil and oxaliplatin)
- Coped very well with treatment
- Ongoing issues: low grade persistent peripheral neuropathy without functional limitation
- Current follow up with medical oncology team 3 monthly and surgeon annually
- Normal colonoscopy last year (12 months post diagnosis)
- Comorbidities: acute myocardial infarct 20 years ago, hypertension, dyslipidaemia, obesity, type 2 diabetes mellitus
- Little regular exercise as he is concerned about his stoma
- Retired academic, lives with wife
- No family history
- Ex-smoker, occasional alcohol use

In plain English

James, a 75 year old man, had rectal cancer 2 years ago after his GP found he had low iron levels. He had chemotherapy and radiotherapy before surgery before the rectum was removed in an operation. The bowel was brought out permanently onto the skin of the abdominal wall and bowel motions now pass through a stoma into a bag. After his operation he then had further chemotherapy.

He was very well during the treatment. He has some ongoing tingling and numbness in the fingertips and toes, but this does not affect him much.

He sees the surgeon once yearly and medical oncology team 3 monthly. His last colonoscopy 12 months ago showed no recurrent cancer.

Although he has had no real problems with his stoma he is worried about it so he does not get out or do much exercise. He had a heart attack 20 years ago. He is obese and has high blood pressure, high cholesterol and diabetes.

**Patient scenario 3**

- 43 year old man, diagnosed with high risk stage II colorectal cancer at age 41
- Hemicolectomy and adjuvant chemotherapy with FOLFOX (5-fluorouracil and oxaliplatin)
- Tolerated chemo extremely well, worked throughout treatment
- Peripheral neuropathy during chemo – mild residual symptoms without functional limitation
- Has occasional faecal incontinence but these episodes are less frequent than they were
- Tumour demonstrated microsatellite instability – saw hereditary cancer and found to have Lynch syndrome
- Family history: no known family history cancer, paternal history unknown
- Current follow up with medical oncology team 3 monthly and surgeon annually
- Works in finance, lives with wife and 10year old daughter. Mother and brother alive and well in Ireland
- Ex-smoker but otherwise in good health. No significant comorbidities
- Exercises daily, goes to gym.
- Has seen exercise physiologist

In plain English

Dominic, a 43 year old man, had colorectal cancer 2 years ago. The cancer has grown through the muscle in the bowel, but it has not spread to the lymph nodes. Half of the colon was removed; this was followed by chemotherapy.

He was well and worked during the chemotherapy. He now has some tingling and numbness in the fingertips and toes, but this does not affect him too much. He still has occasional episodes where he can not control his bowels and there is some leaking of his bowel motions.

He was found to have a genetic mutation called Lynch Syndrome, which makes him more at risk of bowel cancer. There is no family history of cancer, but he is unsure of his father’s family history. His mother and brother, living in Ireland, are both alive and well.

He used to play soccer but has stopped. He now goes to the gym. He has no other medical problems. He works in finance, and lives with his wife and 10 year old daughter. He sees the surgeon once yearly and medical oncology team 3 monthly.

**Supplementary 2: Questionnaire**

**Questionnaire for people who have experienced colorectal cancer**

Date of completion_________________

1. Full name ______________
2. Please provide your residential address ______________
3. Contact phone number ______________
4. Contact email _________________
5. What is your current age?

- <29
- 30-39
- 40-49
- 50-59
- 60-69
- >70

1. What was your age when you were diagnosed with colorectal cancer? _______
2. Please provide details of the cancer treatment received and rate your level of satisfaction

| Cancer treatment received  Examples include chemotherapy and surgery | Dates (from start-finish) | | Rate your level of satisfaction | | | | |
| --- | --- | --- | --- | --- | --- | --- | --- |
|  | |  | Very dissatisfied | Dissatisfied | Neither satisfied nor dissatisfied | Satisfied | Very satisfied |
|  | |  |  |  |  |  |  |
|  | |  |  |  |  |  |  |
|  | |  |  |  |  |  |  |
|  | | - |  |  |  |  |  |
|  | | - |  |  |  |  |  |
|  | | - |  |  |  |  |  |
|  | | - |  |  |  |  |  |

1. Please provide details of all the health care professionals that have seen in the last 12 months and rate your level of satisfaction

| Health care professional seen | Number of times seen | Rate your level of satisfaction | | | | |
| --- | --- | --- | --- | --- | --- | --- |
|  |  | Very dissatisfied | Dissatisfied | Neither satisfied nor dissatisfied | Satisfied | Very satisfied |
|  |  |  |  |  |  |  |
|  |  |  |  |  |  |  |
|  |  |  |  |  |  |  |
|  |  |  |  |  |  |  |
|  |  |  |  |  |  |  |
|  |  |  |  |  |  |  |
|  |  |  |  |  |  |  |

1. Please provide details of the resources that you have used since you were diagnosed with colorectal cancer and rate the usefulness of the resources

| Resources that you have used | Year last used | Rate the usefulness of the resource | | | | |
| --- | --- | --- | --- | --- | --- | --- |
| Examples include: Cancer Council, Cancer Institute and Health direct |  | Totally not useful | Mostly not useful | Somewhat useful | Mostly useful | Totally useful |
|  |  |  |  |  |  |  |
|  |  |  |  |  |  |  |
|  |  |  |  |  |  |  |
|  |  |  |  |  |  |  |
|  |  |  |  |  |  |  |
|  |  |  |  |  |  |  |
|  |  |  |  |  |  |  |

**Questionnaire for GPs and cancer specialists**

Date of completion___________________________________

1. Full name __________
2. Please provide the address of principal practice ______________
3. Contact phone number ______________
4. Contact email _________________
5. What is your current age?

- <29
- 30-39
- 40-49
- 50-59
- 60-69
- >70

1. What is your discipline?

- General practice
- Medical oncologist
- Colorectal surgeon
- Colorectal nurse coordinator

1. How many years have you been in practice in your discipline

- 0-4 years
- 5-9 years
- 10-14 years
- 15-19 years
- 20-24 years
- ≥25 years

1. Please provide the number of half-day sessions in practice per week?

- 3-4 half-day sessions per week
- 5-6 half-day sessions per week
- 7-8 half-day sessions per week
- 9-10 half-day sessions per week
- 11-12 half-day sessions per week
- 13-14 half-day sessions per week

1. Please provide an estimate of the average number of people who have experienced colorectal cancer that you have managed in the last 12 months__________

[For GPs only] Please provide an estimate of the average number of people who have experienced colorectal cancer that you have managed over your career _______

1. Please list the issues that you have managed in people who have experienced colorectal cancer and rate your confidence in managing these issues

­­­­­­­­­­­­­­­­­­­­­­­­

| Issue that you have managed | Year last managed | Rate your confidence in managing this issue | | | | |
| --- | --- | --- | --- | --- | --- | --- |
| Examples include issues with patient investigation, management and ongoing care |  | Totally not confident | Mostly not confident | Somewhat confident | Mostly confident | Totally confident |
|  |  |  |  |  |  |  |
|  |  |  |  |  |  |  |
|  |  |  |  |  |  |  |
|  |  |  |  |  |  |  |
|  |  |  |  |  |  |  |
|  |  |  |  |  |  |  |
|  |  |  |  |  |  |  |

1. Please list the resources that you have used to manage people who have experienced colorectal cancer and rate the usefulness of these resources

­­­­­­­­­­­­­­­­­­­­­­­­

| Resources that you have used | Year last used | Rate the usefulness of the resource | | | | |
| --- | --- | --- | --- | --- | --- | --- |
| Examples include: Canrefer, EviQ, Healthdirect and Cancer Council |  | Totally not useful | Mostly not useful | Somewhat useful | Mostly useful | Totally useful |
|  |  |  |  |  |  |  |
|  |  |  |  |  |  |  |
|  |  |  |  |  |  |  |
|  |  |  |  |  |  |  |
|  |  |  |  |  |  |  |
|  |  |  |  |  |  |  |
|  |  |  |  |  |  |  |
